# Evaluation of MRI in the diagnostic accuracy of extrahepatic metastases in neuroendocrine tumors in comparison with the reference standard somatostatin-receptor–PET/CT

**DOI:** 10.3389/fonc.2023.1194152

**Published:** 2023-08-15

**Authors:** Maria Ingenerf, Johannes Rübenthaler, Vera Wenter, Mathias Zacherl, Friederike Völter, Michael Winkelmann, Homeira Karim, Regina Schinner, Jens Ricke, Frank Berger, Christine Schmid-Tannwald

**Affiliations:** ^1^ Department of Radiology, Ludwig-Maximilians-Universität (LMU) University Hospital, Ludwig-Maximilians-Universität (LMU) Munich, Munich, Germany; ^2^ Department of Nuclear Medicine, Ludwig-Maximilians-Universität (LMU) University Hospital, Ludwig-Maximilians-Universität (LMU) Munich, Munich, Germany; ^3^ European Neuroendocrine Tumor Society (ENETS) Centre of Excellence, Interdisciplinary Center of Neuroendocrine Tumours of the GastroEnteroPancreatic System at at the University Hospital of Munich (GEPNET-KUM), Ludwig-Maximilians-Universität (LMU) University Hospital, Ludwig-Maximilians-Universität (LMU) Munich, Munich, Germany

**Keywords:** neuroendocrine tumors, diffusion magnetic resonance imaging, diagnostic imaging, sensitivity and specificity, accuracy

## Abstract

**Purpose:**

The aim of this study was to compare the diagnostic performance of different sets of MR sequences in detecting extrahepatic disease of NETs on routine liver magnetic resonance imaging (MRI).

**Method:**

One hundred twenty-seven patients with NETs with and without hepatic and extrahepatic metastases who underwent liver MRI and SSTR-PET/CT were retrospectively analyzed. Two radiologists evaluated in consensus in four sessions: (1) non-contrast T1w+T2w (NC), (2) NC+DWI, (3) NC+ contrast-enhanced T1w (CE), and (4) NC+DWI+CE the presence and number of metastases (lymph nodes, bone, peritoneal surface, lung base, and abdominal organ). Sensitivity, specificity, positive, and negative predictive value for detection of metastases were calculated for each session in a patient-based manner; detection and error rates were calculated for lesion-based analysis. Comparison between the MR-sessions and positron emission tomography–computed tomography (PET/CT) was performed with the McNemar test.

**Results:**

Regarding all 1,094 lesions detected in PET/CT, NC+DWI, and NC, CE+DWI identified most true-positive lesions 779 (71%) and 775 (71%), respectively. Patient-based analysis revealed significantly higher sensitivity by NC+DWI (85%) than NC and NC+CE (*p* = 0.011 and 0.004, respectively); the highest specificity was reached by NC+CE+DWI (100%). Site-based analysis revealed highest detection rates for lymph node metastases for NC+DWI and NC, CE+DWI (73 and 76%, respectively); error rates were lower for NC, CE+DWI with 5% compared with 17% (NC+DWI). Detection rates for bone metastases were similarly high in NC+DWI and NC, CE+DWI (75 and 74%, respectively), while CE showed no benefit. For peritoneal metastases highest sensitivity was reached by NC+DWI (67%).

**Conclusion:**

The combination of NC+DWI showed better sensitivities than the combination of NC+CE. NC+DWI showed similar, sometimes even better sensitivities than NC+CE+DWI, but with lower specificities.

## 1 Introduction

Neuroendocrine tumors (NETs) are often well differentiated and show indolent tumor growth. Since NETs express up to 95% somatostatin receptors (SSTRs), the introduction of SSTR PET/CT represented an important improvement in the diagnosis of NETs (1). Imaging with ^68^Ga-DOTA somatostatin analogs is the current recommended modality for staging and re-staging of well-differentiated NET patients and shows in particular high detection rates for lymph node and bone metastases, as well as peritoneal lesions and unknown primary tumors (1, 2). Magnetic resonance imaging on the other hand is the modality of choice for assessing the presence of liver metastases due to better soft-tissue contrast (3). The detection and characterization of liver lesions can be further increased by using liver-specific contrast agents. Thus, MRI is integrated as a complement to PET/CT in the (re)-staging of patients with NET in clinical routine.

Following the ENETS guidelines and the AWMF S2k guideline postoperative follow-up for NET patients is recommended for at least 15 years, which includes MRI liver/abdomen and SSTR-based PET/CT alternating at certain time intervals (1). So, follow-up imaging of NET patients is much more intensive compared with other tumor entities such as pancreatic or colorectal carcinoma, and the patient collective includes many relatively young patients. Against this background, the cumulative radiation exposure should be kept as low as possible to avoid increased secondary malignancies due to intensive follow-up with PET/CT scans.

Especially in low-risk patients, it would be interesting to implement whole-body MRI as an alternative or in addition to PET/CT to rule out metastases in the long-term risk-adapted tumor follow-up. Whole-body MRI is increasingly evaluated for staging various tumor entities (e.g., breast carcinoma or melanoma), with promising results considering the diagnostic accuracy (4, 5). Due to the technical development of MRI in recent years, the image quality has improved, while acquisition times were reduced, which allows the integration of whole-body MRI protocols into clinical routine.

The examination area of liver MRI includes the base of the lungs and the entire upper abdomen (liver, spleen, and pancreas) up to the kidneys. Diffusion-weighted sequences (DWI) are routinely included in the upper abdomen MRI protocol, since it can improve the detection and characterization of liver lesions but seems to be also helpful in detecting other hematogenous or lymphogenous metastases (6–8).

The evaluation of liver MRI includes not only the assessment of hepatic metastases but also evaluation regarding the presence of extrahepatic metastases.

Therefore, it is of great interest to assess the diagnostic accuracy of different sequences integrated in the routine liver MRI protocol in patients with NET compared with the gold standard PET/CT to improve clinical routine by a better understanding of extrahepatic findings in liver MRI and to prepare the groundwork for the development of an MRI whole-body protocol for patients with NET.

## 2 Materials and methods

This study is a retrospective cohort analysis. Patients with neuroendocrine tumors who were examined between 2010 and 2021 with liver MRI with liver-specific contrast agent and ^68^Ga -DOTATATE (-TOC) or ^18^F-SIFA-TATE PET/CT with a maximum interval of 3 months between the two imaging modalities were included. Exclusion criteria were severe imaging artifacts and a missing acquisition of DWI. Approval by the local research ethics committee was obtained, and need for written informed patient consent was waived.

### 2.1 MRI

MR examinations were performed on a 1.5 T MR system (Magnetom Avanto, Magnetom Aera Siemens Healthineers, Erlangen, Germany and Ingenia S, Philips Healthcare, Hamburg, Germany) using a phased-array-coil for signal reception.

The standard imaging protocol consisted of unenhanced T1w gradient-echo (GRE) sequences in- and out-of-phase, single-shot T2w sequence with and without fat suppression (fs) axial and coronar, T1w 3D GRE sequence with fs before and 20, 50, and 120 s after intravenous contrast injection (Gd-EOB-DTPA; Primovist, Eovist, Bayer Pharma, Germany; 25 µmol/kg body weight), multishot T2w turbo spin echo sequence fs, diffusion-weighted sequences with *b*-values of 50, 400, and 800 s/mm² after a delay of 15 min, T1w GRE sequence fs, and an fs T1w 3D GRE sequence.

Detailed sequence parameters are provided in **Table 1**.

**Table 1:** Sequence parameters.

Sequence and parameters	T2w SSFSE Single-shot fast spin echo	DW-MRI	T1w GRE pre- & dynamic post-contrast	T1w GRE sequences in-/out-of-phase	Multishot T2w turbo spin echo sequence
Parallel imaging factor	2	2	2	2	2
Fat saturation	Tra (yes and no) cor (no)	Yes	Yes	No	Yes
Respiratory state	Free breathing	Respiratory gated	Inspiration	Inspiration	Respiratory gated
TR (ms)	800	2800 **(2300)**	3.35	120/110	2860
TE (ms)	84 **(54)**	66 **(70)**	1.19	4.76/2.50	107
FA (deg)	180	180	15	70	180
FOV	380 mm **(380 mm)**	400 mm **(400 mm)**	360 mm **(400 mm)**	380 mm **(380 mm)**	380 mm **(380 mm)**
Matrix	320x320 **(320x189)**	192x130 **(192x113)**	256x154	320 x 168	320 x 180
Slice orientation	Transverse/cor	Transverse	Transverse	Transverse	Transverse
Slice thickness (mm)	6 mm	6 mm	3 mm	6 mm	6 mm
NEX	1	1	1	1	1
No. of slices	35	30	64 **(56)**	72	35
Bandwidth(Hz/pixel)	710 **(446)**	1370	450	450	220
Acquisition time	***	***	18-20s	16-20s	***
b-value (s/mm^2^)	–	50, 800	–	–	–

***Acquisition time depends on the individual patient’s respiratory rate.

Parameters of Magnetom Avanto 1.5 T deviating from Magnetom Aera in bold and brackets.

Tra, transversal; cor, coronal; TR, repetition time; TE, echo time; FA, flip angle; NEX, number of excitations; Hz, Hertz.

### 2.2 PET/CT

^68^Ga-DOTATATE (-TOC) and ^18^F-SIFA-TATE PET/CT were prepared as described previously (9, 10). Whole-body PET/CT scans were acquired in three-dimensional mode (3 min per bed position) using a GE Discovery 690 (GE Healthcare, Little Chalfont, United Kingdom) or a Biograph 64 TruePoint PET/CT scanner (Siemens Healthcare, Erlangen, Germany). Imaging was started 60 min after intravenous administration of around 220 MBq ^68^Ga-DOTA-TATE, (-TOC) or ^18^F-SIFA-TATE, and if possible 20 mg of furosemide. PET/CT scans were performed with a diagnostic CT scan of the neck, thorax, abdomen, and pelvis (100–190 mAs, 120 kV, collimation 2 × 5 mm, pitch of 1.5) and intravenous injection (2.5 mL/s) of an iodine-based contrast agent (Ultravist 300TM; Bayer Healthcare, Berlin, Germany; 1.5 mL/kg body weight) with a delay of 50 s in order to depict the portal venous phase of the liver. Emission data were reconstructed with attenuation correction using concurrent diagnostic CT.

### 2.3 Image analysis

MRI scans were reviewed in four different sessions by two abdominal radiologists in consensus (5 and 12 years of experience, respectively) for identification (number and location) of possible lymphadenopathies, peritoneal, and distant metastases. Multiple and diffuse uncountable lesions were arbitrarily classified as 10 lesions. If disseminated disease was detected an arbitrary number of 10 was assigned. This refers not to ill-defined/conglomerate lesions but rather to multiple small lesions. Both readers were blinded to all information regarding clinical, laboratory, surgical, and pathological findings. The readers were also blinded to the PET/CT findings during the MR reading.

First session (NC) included

- unenhanced T1w GRE sequences in- and out-of-phase
- T1w 3D GRE sequence with fs
- single-shot T2w sequence with and without fs axial and coronar
- multishot T2w turbo spin echo sequence with fs

Second session (NC+DWI) included

- sequences of the first session
- diffusion-weighted sequences with *b*-values of 50 and 800 s/mm²

Third session (NC+CE) included

- sequences of the first session
- T1w 3D GRE sequence with fs 20, 50, and 120 s after intravenous contrast injection (Gd-EOB-DTPA; Primovist, Eovist, Bayer Pharma, Germany; 25 µmol/kg body weight),
- after a delay of 15-min fs T1w GRE axial

Fourth session (NC+DWI+CE) included the combination

- sequences of the third session
- diffusion-weighted sequences with *b*-values of 50, 400, and 800 s/mm²

Lymph node metastases were qualitatively assessed based on shape (round instead of oval were considered malignant), abnormal contrast enhancement indicating presence of necrosis or cystic change and higher b 800 signal intensity than the surrounding lymph nodes. Peritoneal metastases were defined as presence of nodular, confluent, or infiltrative lesions involving the peritoneum, omentum or mesentery with low-signal intensity on T1w and slightly hyperintense on T2w images, with b 800 hyperintensity and contrast enhancement over the peritoneal surfaces, omentum, or mesentery. Distant metastases were defined as nodular, infiltrative, or confluent lesions with low-signal intensity on T1w and slightly hyperintense on T2w images, with b 800 hyperintensity (second reading) and contrast enhancement (11).

### 2.4 Reference standard

SSTR-PET/CT was the primary reference standard. If correlation was impossible, or findings doubtful or in the event that lesions were found on MRI that were not positive on PET/CT, imaging follow-up for at least 6 months was used. PET/CTs were compared by the same radiologists lesion by lesion with the MRI findings.

### 2.5 Statistics

Statistical analysis was performed with IBM SPSS Statistics for Windows, Version 22.0. Armonk, NY; USA: IBM Corp and SAS (version 9.4) for Windows, Cary, NC: SAS Institute Inc. and SAS (version 9.4) for Windows (SAS Institute, Inc.). For data analysis, a significance level of *p* ≤ 0.05 was used. Analysis of the four reading sessions was performed in a lesion- and patient-based manner. For lesion-based evaluation, lesions were classified as true-positive or false-positive and detection rates (TP/all positive lesions according to reference standard) and error rates (FP/all detected lesions). For the patient-based analysis, patients were categorized as positive (with metastatic lesions, independent of exact number) or negative (without metastatic lesions) for predefined organ regions. For patient-based analysis sensitivity, specificity, positive predictive value (PPV), negative predictive value (NPV), and accuracy were calculated with their 95% confidence intervals (CIs). Comparison of sensitivities between the different reading sessions was performed using the exact McNemar test.

## 3 Results

### 3.1 Study population

We included a total of 127 patients [median age: 70; interquartile range (IQR): 63–78; female: 47 (37%)]. Main primary tumor sites were gastrointestinal tract (82 of 127) and pancreas (20 of 127) followed by lung (15 of 127), CUP (seven of 127) and breast (one of 127), kidney (one of 127), and liver (one of 127). Primary tumor was resected in 69% of included patients. Detailed patient characteristics are presented in **Table 2**. No predisposing mutations (e.g., MEN1) were present in the study population. All patients received both a PET/CT and an MRI—107 patients as part of a follow-up and 20 patinets as part of initial staging. Median time interval between liver MRI and the PET/CT as standard of reference was 7 days (IQR: 0–35 days). Sixty-nine of 127 patients underwent ^68^Ga-DOTATOC PET/CT, 38 of 127 patients ^68^Ga-DOTATATE PET/CT, and 20 of 127 patients ^18^F-SIFA-TATE PET/CT.

**Table 2:** Patients´ characteristics.

Characteristic	Value	Percentage (%)
Sex		
Male	80/127	63
Female	47/127	37
Mean age (years)	70 IQR: 63–78	
Primary tumor site		
GI-tract	82/127	64.6
Pancreas	20/127	15.7
Lung	15/127	11.8
CUP	7/127	5.5
Breast Kidney Liver	1/127 1/127 1/127	0.8 0.8 0.8
Grading		
G1	51/127	40.2
G2	56/127	44.1
G3	6/127	4.7
n/a	14/127	11.0
Ki-67		
≤ 2%	33/127	26
> 2–20%	70/127	55.1
> 20% n/a	6/127 18/127	4.7 14.2

According to the standard of reference 77% (98/127) of patients had extrahepatic disease (including primary tumor). A total number of 1,094 extrahepatic lesions was found in PET scans. These included mainly lesions classified as bone metastases (*n* = 611), metastatic lymph nodes (*n* = 326), and peritoneal metastases (*n* = 106), as well as a smaller percentage of lesion representing metastasis to other abdominal organs (*n* = 36) and lung (*n* = 15). Detailed numbers of the specific tumor sites (e.g., retroperitoneal or mesenterial lymph nodes) are presented in **Table 3**. On a patient-based level 83% (*n* = 105) showed hepatic metastases, 59% (*n* = 75) bone metastases, 49% (*n* = 62) lymphatic metastases, 24% (*n* = 30) peritoneal metastases, 6% (*n* = 8) pulmonal metastases and 27% (*n* = 34) other abdominal organ metastases in the depicted range of the upper abdomen images.

**Table 3:** Lesions found in standard of reference (PET/CT).

Location	Number	Percentage (%)
**Lymph nodes**	326	29.8
Retroperitoneal	177	16.2
Mesenterial	113	10.3
Cardiophrenic	36	3.3
**Bone**	611	55.9
Spine	453	41.4
Rib Cage	158	14.4
**Peritoneal**	106	9.7
Subphrenic	16	1.5
Paracolic	11	1.0
Gastric serosa	7	0.6
Free peritoneal surface	63	5.8
Gerota fascia	9	0.8
**Lung**	15	1.4
**Other abdominal organs**	36	3.3
Adrenal gland	2	0.2
Pancreas	23	2.1
Spleen	5	0.5
GIT	6	0.5
**Total**	1094	

### 3.2 Lesion-based analysis

Regarding all 1,094 lesions detected in PET/CT, the combination of NC+DWI and NC+DWI+CE identified most true-positive lesions with a total number of 779 (71%) and 775 (71%), respectively. NC+ CE found more true-positive lesions than NC with 592 (54%) lesions compared with 482 (44%). Total error rates among the four readings were comparable, ranging from 7% (NC+CE) to 10% (NC+DWI).

**Figure 1:**
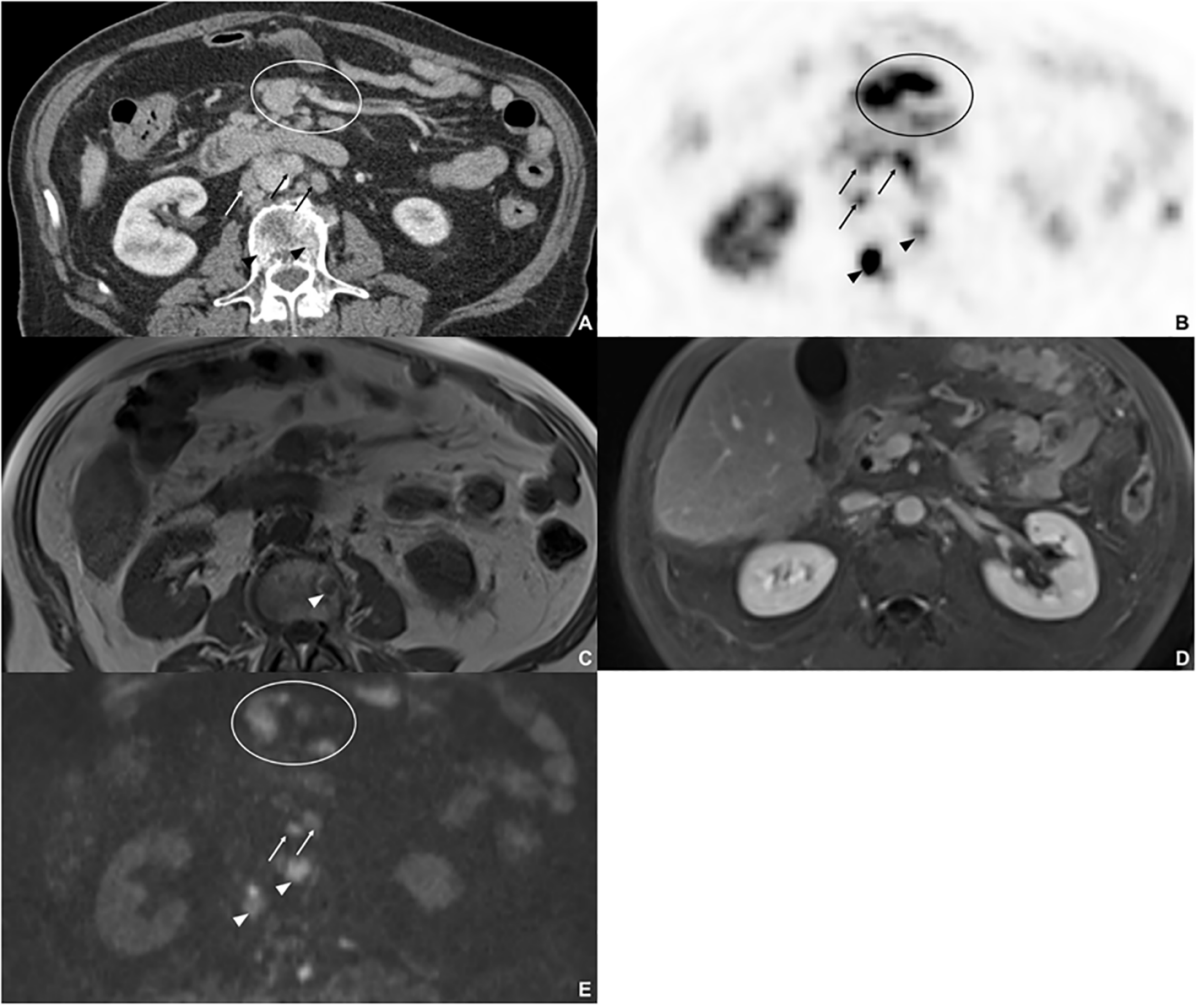
Eighty-five-year-old male with metastatic NET, originating from the gastrointestinal tract. On ^68^Ga-DOTATATE PET/CT **(A, B)**. two sclerotic bone lesions (arrow head), three retroperitoneal lymph node metastases, and mesenterial tumor manifestation with high uptake were detected. On non-contrast T1w image **(C)**, one sclerotic metastasis with low-signal intensity was detected. On the axial contrast-enhanced T1w image **(D)**, no metastases and tumor manifestation were detected, since there was no enhancement of the bone metastases, the lymph node metastases were not rated as metastases due to size and oval shape and mesenterial tumor manifestation was misinterpreted as intestine. On DWI **(E)**, both bone marrow metastases were detected, two of the retroperitoneal lymph node metastases and the mesenteric tumor manifestation.

Site-based analysis revealed highest detection rates for lymph node metastases for NC+DWI and NC+DWI+CE with 73 and 76%, respectively; however, error rate was lower for NC+DWI+CE with 5% compared with 17% with NC+DWI (**Figure 1**). Subsite analysis showed that NC+DWI and NC + DWI + CE showed similarly good detection rates for retroperitoneal and mesenterial lymph node metastases, while cardiophrenic lymph node metastases were best found in NC+DWI+CE and NC+CE (detection rates 78 and 69%, respectively).

**Figure 2:**
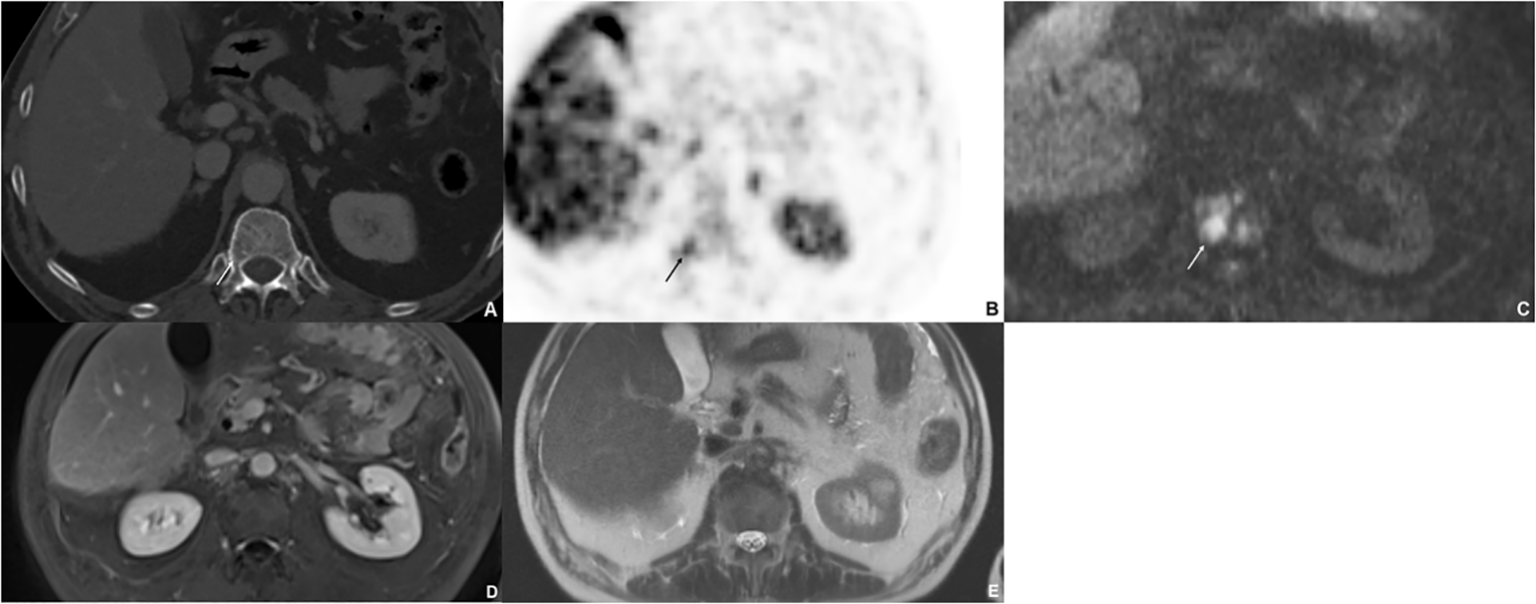
Sixty-nine-year-old male with bone metastases of gastrointestinal NET. The CT **(A)** shows a lytic lesion (arrow) that is difficult to define; however, the bone metastasis shows a significant uptake ^18^F-SIFA-TATE PET/CT **(B)**. The lesion is strongly diffusion restricted **(C)**; however, it was not visible on postcontrast T1w image **(D)** nor on T2w image **(E)**.

Detection rates for bone metastases (**Figures 1**, **2**) were highest in NC+DWI and NC+DWI+CE (75 and 74%, respectively). Metastases in the spine were detected well with both mentioned reading sets (82 and 78%, respectively), while detection rates of skeletal metastases in the rib cage were lower with comparable performances in NC+DWI and NC+DWI+CE (55 and 63%, respectively).

**Figure 3:**
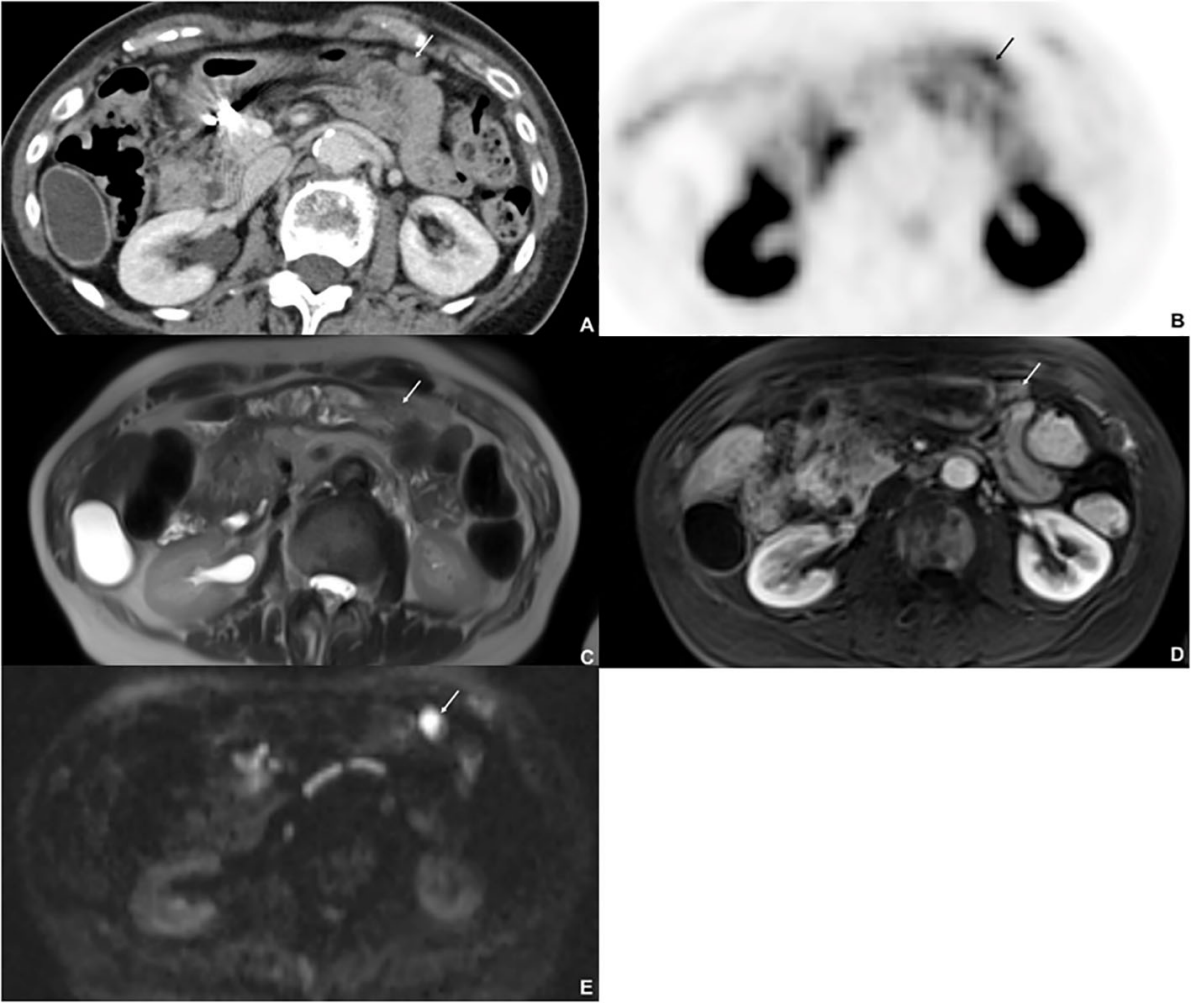
Seventy-seven-year-old female with metastatic NET, originating from the lung. ^68^Ga-DOTATATE PET/CT shows a peritoneal lesion on CT **(A)** (arrow) at the anterior peritoneal surface with high uptake **(B)**. The lesion was missed on T2w image **(C)** and on postcontrast T1w image (arterial phase) **(D)** but clearly detected on DWI **(E)**: The lesion shows restricted diffusion.

For peritoneal metastases detection rates were rather low to average in all readings and were best found in NC + DWI and NC+DWI+CE (56 and 52%) (**Figure 3**). Regarding subsites peritoneal lesions were best detected when located subphrenic or at the gerota fascia (detection rates of 63% and 78% in NC + DWI) while detection rates were lowest for lesions located at the gastric serosa (best 29% in NC + DWI + CE).

Detection rates for lung metastases were low for all four readings (33 – 40%), while detection rates for other abdominal organ lesions were especially high for pancreatic lesions in NC+DWI and NC+DWI+CE with 78 and 87%, respectively. A detailed overview of the lesion-based analysis including subsites is depicted in **Tables 4**, **5**.

**Table 4:** Lesion based comparison for organ involvement.

Organ involvement	No. of lesions (PET/CT)	Sequences	TP	FP	Detection rate	Error rate
Lymph nodes	326	NC	137	27	0.42	0.16
		NC+DWI	237	48	0.73	0.17
		NC+CE	210	14	0.64	0.06
		NC+DWI+CE	247	12	0.76	0.05
Bone	611	NC	293	8	0.48	0.03
		NC+DWI	457	13	0.75	0.03
		NC+CE	322	11	0.53	0.03
		NC+DWI+CE	450	52	0.74	0.10
Peritoneal	106	NC	33	6	0.31	0.15
		NC+DWI	59	22	0.56	0.27
		NC+CE	41	14	0.39	0.28
		NC+DWI+CE	55	10	0.52	0.15
Lung	15	NC	5	1	0.33	0.17
		NC+DWI	6	1	0.40	0.14
		NC+CE	5	1	0.33	0.17
		NC+DWI+CE	5	1	0.33	0.17
Other organs	36	NC	16	6	0.44	0.27
		NC+DWI	26	4	0.72	0.13
		NC+CE	17	5	0.47	0.23
		NC+DWI+CE	27	1	0.75	0.04

**Table 5:** Lesion based comparison with subsite analysis.

Organ involvement	No. of lesions (PET/CT)	Sequences	TP	FP	Detection rate	Error rate
Lymph nodes						
Retroperitoneal	177	NC	104	7	0.59	0.06
		NC+DWI	141	15	0.80	0.10
		NC+CE	122	8	0.69	0.06
		NC+DWI+CE	139	3	0.79	0.02
Mesenterial	113	NC	23	12	0.20	0.34
		NC+DWI	80	26	0.71	0.25
		NC+KM	63	2	0.56	0.03
		NC+DWI+CE	80	5	0.71	0.06
Cardiophrenic	36	NC	10	8	0.28	0.44
		NC+DWI	16	7	0.44	0.30
		NC+CE	25	4	0.69	0.14
		NC+DWI+CE	28	4	0.78	0.13
Bone						
Spine	453	NC	289	7	0.64	0.02
		NC+DWI	370	5	0.82	0.01
		NC+CE	284	6	0.63	0.02
		NC+DWI+CE	350	22	0.77	0.06
Rib Cage	158	NC	4	1	0.03	0.20
		NC+DWI	87	8	0.55	0.08
		NC+CE	38	5	0.24	0.12
		NC+DWI+CE	100	30	0.63	0.23
Peritoneal						
Subphrenic	16	NC	3	0	0.19	0.00
		NC+DWI	10	0	0.63	0.00
		NC+CE	4	10	0.25	0.71
		NC+DWI+CE	10	1	0.63	0.09
Paracolic	11	NC	3	1	0.27	0.25
		NC+DWI	4	10	0.36	0.71
		NC+CE	3	0	0.27	0.00
		NC+DWI+CE	4	1	0.36	0.20
Gastric serosa	7	NC	1	1	0.14	0.50
		NC+DWI	1	2	0.14	0.67
		NC+CE	1	1	0.14	0.33
		NC+DWI+CE	2	0	0.29	0.00
Free peritoneal surface	63	NC	21	1	0.33	0.05
		NC+DWI	37	3	0.59	0.08
		NC+CE	30	0	0.48	0.00
		NC+DWI+CE	34	2	0.54	0.06
Gerota fascia	9	NC	5	3	0.56	0.38
		NC+DWI	7	7	0.78	0.50
		NC+CE	3	3	0.33	0.50
		NC+DWI+CE	5	6	0.56	0.55
Other abdominal organs						
Adrenal gland	2	NC	1	1	0.50	0.50
		NC+DWI	2	0	1.00	0.00
		NC+CE	1	0	0.50	0.00
		NC+DWI+CE	1	0	0.50	0.00
Pancreas	23	NC	11	1	0.48	0.08
		NC+DWI	18	1	0.78	0.05
		NC+CE	13	3	0.57	0.19
		NC+DWI+CE	20	1	0.87	0.05
Spleen	5	NC	3	3	0.40	0.67
		NC+DWI	3	3	0.60	0.50
		NC+CE	2	1	0.40	0.33
		NC+DWI+CE	3	0	0.60	0.00
GIT	6	NC	2	0	0.33	0.00
		NC+DWI	3	0	0.50	0.00
		NC+CE	1	1	0.17	0.50
		NC+DWI+CE	3	0	0.50	0.00

### 3.3 Patient-based analysis

Regarding patients with extrahepatic metastases detected in PET/CT, the highest sensitivity was reached by the combination of NC+DWI (85%), which was significantly higher compared with NC only and compared with NC +CE (*p* = 0.011 and 0.004, respectively); however, the highest specificity was reached by combining NC + DWI + CE (1.00).

For lymph node metastases, sensitivity was significantly higher in NC+DWI than in the other readings (*p* < 0.03) with 77%, while specificity was only slightly, but not significantly lower than in the other sets of sequences.

In the assessment of bone metastases, the addition of DWI significantly increased sensitivity compared with non-enhanced imaging and NC+CE (*p* < 0.005), while there was no significant difference between in NC+DWI and NC+DWI+CE. Sensitivities for peritoneal metastases were highest in NC+DWI and the addition of DWI was significantly better than NC+CE (*p* = 0.0005).

Sensitivities and specificities for pulmonal metastases were rather low in all readings, and there were no significant differences between reading sets.

For the assessment of metastatic/primary lesions of other abdominal organs, the addition of DWI slightly improved sensitivity compared with non-enhanced reading (*p* < 0.02) but not compared with NC+CE. A detailed summary of the patient-based analysis is presented in **Table 6**.

**Table 6:** Patient-based comparison for organ involvement.

Organ	Reading	TP	FP	Sensitivity	95% CI	Specificity	95% CI	PPV	NPV	Accuracy
Overall extrahepatic	NC	73	6	0.74	0.65–0.83	0.70	0.60–0.92	0.92	0.48	0.76
	NC+DWI	84	5	0.86	0.77–0.92	0.83	0.64–0.94	0.94	0.63	0.85
	NC+CE	70	3	0.71	0.61–0.8	0.90	0.73–0.98	0.96	0.48	0.76
	NC+DWI+CE	77	0	0.79	0.69–0.86	1.0	0.88–1.0	1.0	0.58	0.83
Lymph nodes	NC	33	7	0.53	0.4–0.66	0.89	0.79–0.96	0.83	0.67	0.72
	NC+DWI	48	11	0.77	0.65–0.87	0.83	0.72–0.91	0.81	0.79	0.80
	NC+CE	34	3	0.55	0.42–0.68	0.95	0.87–0.99	0.92	0.69	0.76
	NC+DWI+CE	40	4	0.65	0.51–0.76	0.94	0.85–0.98	0.91	0.73	0.80
Bone	NC	47	3	0.64	0.51–0.74	0.94	0.84–0.99	0.94	0.65	0.76
	NC+DWI	61	4	0.81	0.71–0.89	0.92	0.81–0.98	0.94	0.79	0.87
	NC+CE	46	5	0.61	0.49–0.72	0.90	0.79–0.97	0.90	0.62	0.73
	NC+DWI+CE	52	3	0.69	0.58–0.79	0.94	0.84–0.99	0.95	0.68	0.80
Peritoneal	NC	11	3	0.37	0.2–0.56	0.97	0.91–1.0	0.79	0.83	0.83
	NC+DWI	20	3	0.67	0.47–0.83	0.97	0.91–1.0	0.87	0.90	0.90
	NC+CE	6	1	0.20	0.08–0.39	1.00	0.96–1.0	0.86	0.80	0.80
	NC+DWI+CE	15	5	0.50	0.31–0.68	0.95	0.88–0.98	0.75	0.86	0.84
Lung	NC	3	1	0.38	0.09–0.76	0.99	0.95–1.0	0.75	0.95	0.94
	NC+ DWI	4	1	0.50	0.16–0.84	0.99	0.95–1.0	0.80	0.97	0.96
	NC+CE	2	1	0.25	0.03–0.65	0.99	0.95–1.0	0.67	0.95	0.94
	NC+DWI+CE	2	1	0.25	0.03–0.65	0.99	0.95–1.0	0.67	0.95	0.94
Other abdominal organs	NC	14	3	0.41	0.22–0.56	0.97	0.91–0.99	0.82	0.82	0.82
	NC+DWI	21	1	0.62	0.44–0.78	0.99	0.94–0.99	0.95	0.88	0.89
	NC+CE	15	3	0.44	0.27–0.62	0.97	0.91–0.99	0.83	0.83	0.83
	NC+DWI+CE	17	2	0.50	0.32–0.68	0.98	0.92–1.0	0.89	0.84	0.85

## 4 Discussion

In the present study, lesion- and patient-based diagnostic performance of different MRI sequence combinations of routine liver- for the detection of extrahepatic disease of patients with NET was evaluated.

In total, the highest lesion-based detection rates were found by combining NC+DWI or NC+DWI+CE with 71% each. Concordantly, highest overall sensitivities for the presence of extrahepatic metastases in the patient-based analysis were reached by the combination of NC+DWI (85%) and NC+DWI+CE (78%), with higher specificity when combining all available sequences (100%). Regarding the extrahepatic disease pattern, we found especially good results for the detection of bone and lymphatic metastasis with the addition of DWI only (81 and 77%, respectively) as well as for pancreatic tumor manifestations (87%) when adding both DWI and CE.

Schraml et al. reported higher sensitivities (98%) for overall patient-based assessment of metastatic status comparing the diagnostic performance for ^68^Ga-DOTATOC PET/CT and whole-body MRI (wbMRI). However, the authors also assessed the presence of liver metastases, which constituted a high proportion of all analyzed lesions (12). Since MRI represents the best modality for hepatic metastases, due to higher soft tissue-contrast and the availability of liver-specific contrast agents, higher overall performance in their study was not surprising. By contrast, the focus of our work was a better characterization of the potential of MRI for extrahepatic disease. Schraml et al. reported significantly higher sensitivities of PET/CT for metastasis of lymph nodes (100 vs. 73%) and lungs (100 vs. 87%), while wb MRI showed higher detection rates for bone metastases (96 vs. 82%) (12). By comparison, we reached a slightly higher sensitivity (77%) for the detection of lymph node metastases using only DWI in addition to the non-contrast enhanced sequences, which was significantly higher than sensitivities in the other readings. Subsite analysis revealed that the integration of DWI to the reading led to high detection rates for retroperitoneal and mesenterial lymph node metastases, while for the assessment of cardiophrenic lymph nodes contrast enhanced (NC+DWI+CE and NC+CE) sequence combinations showed improved detection and error rates. However, one must be aware that both normal lymph nodes and metastatic lymph nodes may have a high-signal intensity on DWI (13). In addition, DWI is prone to artifacts due to respiratory and heart movement, so that these results may not be reproducible in the thoracic region. This is underlined by rather low detection rates and high error rates for NC+DWI in the evaluation of cardiophrenic lymphatic metastasis, while the addition of contrast-enhanced sequences led to good detection rates for this region (up to 78%).

Lesion-based detection rates for bone metastases were highest in NC+DWI and NC+DWI+CE (75 and 74%, respectively). In the patient-based assessment of bone metastases, the addition of DWI significantly increased sensitivity compared with non-enhanced imaging and NC+CE. This is in accordance with previous published studies that have shown that MRI including DWI represents an excellent diagnostic method for detection of bone metastases (13–15). Multiple studies suggested that wb MRI with DWI (often without CE) showed similar or even better detection rates than CT or PET/CT for bone metastases of renal cell carcinoma (8), breast cancer (4), and prostate cancer (16, 17). However, it should be noted that bone metastases differ in their appearance, for example sclerotic skeletal metastases show low-signal intensities on MRI while visual contrast is better with PET/CT in these metastases (18–20). Baur et al. showed that sclerotic bone metastases treated with chemotherapy had a hypointense appearance on DWI due to low water content (21). Notably error rates for bone metastasis were not improved by contrast-enhanced sequences in our study.

Overall detection rates of peritoneal metastases turned out to be only mediocre; however, addition of DWI could significantly improve the sensitivity compared with NC+CE. Cianci et al. also showed also that the sensitivity for the detection of peritoneal metastases by combined interpretation of conventional MRI with DWI was significantly higher compared with conventional contrast-enhanced sequences alone (22). In our analysis, peritoneal manifestations were best detected when located subphrenic or at the gerota fascia while detection rates were lowest for lesions located at the gastric serosa or paracolic, which might be explained by respiratory motion artifacts or difficulties in differentiation from colonic diverticula, respectively. When developing a whole-body MRI protocol, sopolamine would probably be useful as a drug to reduce bowel motility. Furthermore, the sensitivity could improve if readers had access to previous PET/CT scans as it would be the setting in clinical routine.

Lesion- and patient-based detection rates for lung metastases were low for all four readings, however these results were expected since MRI is not the modality of choice for assessing lung metastases and a low-dose CT should therefore be considered as a screening supplement to a possible wb MRI.

Evaluation of tumor manifestations in other abdominal organs was limited by small total numbers of lesions apart from pancreatic tumors (*n* = 23) (see **Table 2**). Detection rates for pancreatic NET manifestations were notably improved by the addition of DWI (18 of 23 lesions) compared with NC or NC+CE (11 of 23 or 13 of 23, respectively) and were highest for NC+DWI+CE (20/23). Schmid-Tannwald et al. also reported that the detection of pancreatic NET was significantly increased by DWI as adjunct to T2w imaging and performance was comparable with CE T1w imaging (23).

At first glance, it seems surprising that detection rates with the combination of non-contrast NC+DWI+CE are sometimes lower than those of non-contrast NC+DWI. This might be explained by the fact that when assessing a diffusion-restricted and therefore suspicious lesion, a lack of or only low contrast enhancement leads to this lesion being falsely assessed as benign. This may especially play a role in NET tumor manifestations, since this tumor entity is primarily arterially hypervascular and the arterial phase is particularly susceptible to artifacts, especially with Eovist/Primovist if the injection is too rapid causing transient severe respiratory motion (24). On the other hand, if the lesion is diffusion-restricted and shows the typical contrast uptake, this leads to higher specificity with the combination of non-contrast, contrast-enhanced sequences and DWI compared with the combination of non-contrast-enhanced sequences alone and DWI, as we also found in our study.

Limitation of this study was its retrospective character with some of the patients already undergoing therapy at the time of the analysis, so that possible therapy effects might have influenced our evaluation. Another limitation represents the lack of pathological confirmation as gold standard; however, SSTR-PET/CT is currently the most accurate imaging modality for NET and follow-up imaging was available in case of doubtful findings. However, it is known that NET metastases can lose their SSTR expression during dedifferentiation and were not being taken into account. As the study focused on clinically available liver/upper-abdomen MRIs the range of included field of view varied intra-individually from patient to patient and other field of interests such as neck and thorax were not evaluated in this study. Whole-body MRI might be an interesting alternative for future studies; however, we also wanted to emphasize experience relevant to everyday clinical practiceOne major limitation which warrants mention is that MRI may be detecting disease that PET did not, particularly in lesions that are too small to detect on PET or which were PET-negative lesions. Therefore some of the false positives may in fact be true disease. However, in the event that lesions found on MRI were not positive on PET/CT, follow-up imaging and other imaging techniques were used to form a consensus.

## 5 Conclusion

The combination of NC+DWI showed better overall detection of extrahepatic NET than the combination of NC+CE. The combination of NC+DWI showed similar, sometimes even better sensitivities than NC+DWI+CE, ​​but with slightly lower specificity. Upper-abdomen MRI showed especially good detection rates for bone metastases, lymphatic metastasis, and pancreatic tumors, while the detection of peritoneal metastases was only good for lesions located subphrenic and at the gerota fascia. The addition of intravenous contrast especially improved detection rates for cardiophrenic lymph nodes and pancreatic NET.

These results should be considered in the daily routine when examining liver MRI with regard to extrahepatic metastases and should be taken into account for the development of a wb MRI protocol, since selection of a combination of sequences with the best diagnostic performance are key questions for the implementation of wb MRI into clinical routine (25, 26). However, further research is needed to improve relatively low detection rates in some categories compared with PET/CT despite usually more sequences of contrast enhancement in liver MRI compared with a wb MRI protocol.

## Data availability statement

The raw data supporting the conclusions of this article will be made available by the authors, without undue reservation.

## Ethics statement

The studies involving human participants were reviewed and approved by Ethikkommission bei der LMU München (local ethics committee). Written informed consent for participation was not required for this study in accordance with the national legislation and the institutional requirements.

## Author contributions

All authors contributed to the study conception and design. Material preparation and data collection were performed by MI, FV, HK, MW, and CS-T. Formal analysis was performed by RS and MI. The first draft of the manuscript was written by MI and CS-T and all authors commented on previous versions of the manuscript. All authors contributed to the article and approved the submitted version.

## References

[1] Rinke A Wiedenmann B Auernhammer C Bartenstein P Bartsch DK Begum N. Practice guideline neuroendocrine tumors - awmf-reg. 021-27. Z Gastroenterol (2018) 56(6):583–681. doi: 10.1055/a-0604-2924 29890561

[2] Sadowski SM Neychev V Millo C Shih J Nilubol N Herscovitch P. Prospective study of 68ga-dotatate positron emission tomography/computed tomography for detecting gastro-entero-pancreatic neuroendocrine tumors and unknown primary sites. J Clin Oncol (2016) 34(6):588–96. doi: 10.1200/jco.2015.64.0987 PMC4872030 26712231

[3] Sundin A Arnold R Baudin E Cwikla JB Eriksson B Fanti S. Enets consensus guidelines for the standards of care in neuroendocrine tumors: radiological, nuclear medicine & Hybrid imaging. Neuroendocrinology (2017) 105(3):212–44. doi: 10.1159/000471879 28355596

[4] Bhaludin BN Tunariu N Koh DM Messiou C Okines AF McGrath SE. A review on the added value of whole-body mri in metastatic lobular breast cancer. Eur Radiol (2022) 32(9):6514–25. doi: 10.1007/s00330-022-08714-6 35384456

[5] Jansen YJL Willekens I Seremet T Awada G Schwarze JK De Mey J. Whole-body mri for the detection of recurrence in melanoma patients at high risk of relapse. Cancers (Basel) (2021) 13(3):442. doi: 10.3390/cancers13030442 33503861 PMC7865287

[6] Galea N Cantisani V Taouli B. Liver lesion detection and characterization: role of diffusion-weighted imaging. J magnetic resonance Imaging JMRI (2013) 37(6):1260–76. doi: 10.1002/jmri.23947 23712841

[7] Caglic I Barrett T. Diffusion-weighted imaging (Dwi) in lymph node staging for prostate cancer. Transl Androl Urol (2018) 7(5):814–23. doi: 10.21037/tau.2018.08.04 PMC6212625 30456184

[8] Beuselinck B Pans S Bielen J De Wever L Noppe N Vanderschueren G. Whole-body diffusion-weighted magnetic resonance imaging for the detection of bone metastases and their prognostic impact in metastatic renal cell carcinoma patients treated with angiogenesis inhibitors. Acta Oncol (2020) 59(7):818–24. doi: 10.1080/0284186x.2020.1750696 32297532

[9] Breeman WA de Jong M de Blois E Bernard BF Konijnenberg M Krenning EP. Radiolabelling dota-peptides with 68ga. Eur J Nucl Med Mol Imaging (2005) 32(4):478–85. doi: 10.1007/s00259-004-1702-y 15655678

[10] Ilhan H Lindner S Todica A Cyran CC Tiling R Auernhammer CJ. Biodistribution and first clinical results of (18)F-sifalin-tate pet: A novel (18)F-labeled somatostatin analog for imaging of neuroendocrine tumors. Eur J Nucl Med Mol Imaging (2020) 47(4):870–80. doi: 10.1007/s00259-019-04501-6 31492994

[11] Dresen RC De Vuysere S De Keyzer F Van Cutsem E Prenen H Vanslembrouck R. Whole-body diffusion-weighted mri for operability assessment in patients with colorectal cancer and peritoneal metastases. Cancer Imaging Off Publ Int Cancer Imaging Soc (2019) 19(1):1. doi: 10.1186/s40644-018-0187-z PMC6322317 30616608

[12] Schraml C Schwenzer NF Sperling O Aschoff P Lichy MP Müller M. Staging of neuroendocrine tumours: comparison of [^68^ga]Dotatoc multiphase pet/ct and whole-body mri. Cancer Imaging Off Publ Int Cancer Imaging Soc (2013) 13(1):63–72. doi: 10.1102/1470-7330.2013.0007 PMC3589947 23466785

[13] Carlbom L Caballero-Corbalán J Granberg D Sörensen J Eriksson B Ahlström H. Whole-body mri including diffusion-weighted mri compared with 5-htp pet/ct in the detection of neuroendocrine tumors. Ups J Med Sci (2017) 122(1):43–50. doi: 10.1080/03009734.2016.1248803 27894208 PMC5361431

[14] Gutzeit A Doert A Froehlich JM Eckhardt BP Meili A Scherr P. Comparison of diffusion-weighted whole body mri and skeletal scintigraphy for the detection of bone metastases in patients with prostate or breast carcinoma. Skeletal Radiol (2010) 39(4):333–43. doi: 10.1007/s00256-009-0789-4 20205350

[15] Messiou C Collins DJ Morgan VA Desouza NM. Optimising diffusion weighted mri for imaging metastatic and myeloma bone disease and assessing reproducibility. Eur Radiol (2011) 21(8):1713–8. doi: 10.1007/s00330-011-2116-4 21472473

[16] Shen G Deng H Hu S Jia Z. Comparison of choline-pet/ct, mri, spect, and bone scintigraphy in the diagnosis of bone metastases in patients with prostate cancer: A meta-analysis. Skeletal Radiol (2014) 43(11):1503–13. doi: 10.1007/s00256-014-1903-9 24841276

[17] Jambor I Kuisma A Ramadan S Huovinen R Sandell M Kajander S. Prospective evaluation of planar bone scintigraphy, spect, spect/ct, 18f-naf pet/ct and whole body 1.5t mri, including dwi, for the detection of bone metastases in high risk breast and prostate cancer patients: skeleta clinical trial. Acta Oncol (2016) 55(1):59–67. doi: 10.3109/0284186x.2015.1027411 25833330

[18] Beiderwellen K Huebner M Heusch P Grueneisen J Ruhlmann V Nensa F. Whole-body [^18^f]Fdg pet/mri vs. Pet/ct in the assessment of bone lesions in oncological patients: initial results. Eur Radiol (2014) 24(8):2023–30. doi: 10.1007/s00330-014-3229-3 24907940

[19] Schmidt GP Schoenberg SO Schmid R Stahl R Tiling R Becker CR. Screening for bone metastases: whole-body mri using a 32-channel system versus dual-modality pet-ct. Eur Radiol (2007) 17(4):939–49. doi: 10.1007/s00330-006-0361-8 16951929

[20] Sawicki LM Deuschl C Beiderwellen K Ruhlmann V Poeppel TD Heusch P. Evaluation of (68)Ga-dotatoc pet/mri for whole-body staging of neuroendocrine tumours in comparison with (68)Ga-dotatoc pet/ct. Eur Radiol (2017) 27(10):4091–9. doi: 10.1007/s00330-017-4803-2 28439648

[21] Baur A Dietrich O Reiser M. Diffusion-weighted imaging of bone marrow: current status. Eur Radiol (2003) 13(7):1699–708. doi: 10.1007/s00330-003-1873-0 12759770

[22] Cianci R Delli Pizzi A Patriarca G Massari R Basilico R Gabrielli D. Magnetic resonance assessment of peritoneal carcinomatosis: is there a true benefit from diffusion-weighted imaging? Curr Probl Diagn Radiol (2020) 49(6):392–7. doi: 10.1067/j.cpradiol.2019.06.002 31248709

[23] Schmid-Tannwald C Schmid-Tannwald CM Morelli JN Neumann R Haug AR Jansen N. Comparison of abdominal mri with diffusion-weighted imaging to 68ga-dotatate pet/ct in detection of neuroendocrine tumors of the pancreas. Eur J Nucl Med Mol Imaging (2013) 40(6):897–907. doi: 10.1007/s00259-013-2371-5 23460395

[24] Davenport MS Bashir MR Pietryga JA Weber JT Khalatbari S Hussain HK. Dose-toxicity relationship of gadoxetate disodium and transient severe respiratory motion artifact. AJR Am J roentgenol (2014) 203(4):796–802. doi: 10.2214/ajr.13.11587 25055154

[25] Larbi A Omoumi P Pasoglou V Michoux N Triqueneaux P Tombal B. Whole-body mri to assess bone involvement in prostate cancer and multiple myeloma: comparison of the diagnostic accuracies of the T1, short tau inversion recovery (Stir), and high B-values diffusion-weighted imaging (Dwi) sequences. Eur Radiol (2019) 29(8):4503–13. doi: 10.1007/s00330-018-5796-1 30413957

[26] Messiou C Kaiser M. Whole body diffusion weighted mri–a new view of myeloma. Br J Haematol (2015) 171(1):29–37. doi: 10.1111/bjh.13509 26013304 PMC4737237

